# Performance modulation of *α*-MnO_2_ nanowires by crystal facet engineering

**DOI:** 10.1038/srep08987

**Published:** 2015-03-11

**Authors:** Wenxian Li, Xiangyuan Cui, Rong Zeng, Guodong Du, Ziqi Sun, Rongkun Zheng, Simon P. Ringer, Shi Xue Dou

**Affiliations:** 1 Institute for Superconducting and Electronic Materials, University of Wollongong, NSW 2522, Australia; 2 School of Materials Science and Engineering, Shanghai University, Shanghai 200072, PR China; 3 Solar Energy Technologies, School of Computing, Engineering and Mathematics, University of Western Sydney, Penrith, NSW 2751, Australia; 4 Australian Centre for Microscopy and Microanalysis, The University of Sydney, NSW 2006, Australia; 5 School of Aerospace, Mechanical and Mechatronic Engineering, The University of Sydney, NSW 2006, Australia; 6 School of Physics, The University of Sydney, Sydney, NSW 2006, Australia

## Abstract

Modulation of material physical and chemical properties through selective surface engineering is currently one of the most active research fields, aimed at optimizing functional performance for applications. The activity of exposed crystal planes determines the catalytic, sensory, photocatalytic, and electrochemical behavior of a material. In the research on nanomagnets, it opens up new perspectives in the fields of nanoelectronics, spintronics, and quantum computation. Herein, we demonstrate controllable magnetic modulation of *α*-MnO_2_ nanowires, which displayed surface ferromagnetism or antiferromagnetism, depending on the exposed plane. First-principles density functional theory calculations confirm that both Mn- and O-terminated *α*-MnO_2_ (1 1 0) surfaces exhibit ferromagnetic ordering. The investigation of surface-controlled magnetic particles will lead to significant progress in our fundamental understanding of functional aspects of magnetism on the nanoscale, facilitating rational design of nanomagnets. Moreover, we approved that the facet engineering pave the way on designing semiconductors possessing unique properties for novel energy applications, owing to that the bandgap and the electronic transport of the semiconductor can be tailored via exposed surface modulations.

The morphology related properties of nanomaterials have attracted growing research interest for generating peculiar properties with great potential for practical innovative applications^1^,^2^,^3^,^4^,^5^. Crystal facet engineering is known to induce exotic physical and chemical performance in functional materials due to the distorted electronic structure and different exposed ions in the surface layers of inorganic crystals with different exposed planes^6^,^7^,^8^,^9^. Scientific and technological exploration has shown the profound influence of such surface layer in research on catalysis and photocatalysis. Xie *et al.* found that the (1 1 0) facet exposed Co_3_O_4_ nanorods had the ability to catalyze CO oxidation at temperatures as low as 77K, because the (1 1 0) planes expose active Co^3+^ species at the surface and allow the CO oxidizes at Co^3+^ sites at such a low temperature^6^. In research on photocatalysis, anatase TiO_2_ showed promise for energy and environmental applications if the active (0 0 1) planes were exposed on the surface^7^. Tian et al. synthesized platinum nanocrystals with an unusual tetrahexahedral shape, with the polyhedra enclosed by 24 high-index facets, such as (7 3 0) and (5 2 0) surfaces with high density of atomic steps and dangling bonds. These surfaces exhibit enhanced catalytic activity compared to equivalent conventional Pt surfaces towards electro-oxidation of small organic molecules such as ethanol and formic acid^8^. Thereafter, Zhang et al. investigated the catalytic reaction processes of triiodide reduction over {1 0 0}, {1 1 1} and {4 1 1} facets of Pt, indicating that the activity follows the order of Pt(1 1 1) > Pt(4 1 1) > Pt(1 0 0) using density functional theory^9^. The highest photovoltaic conversion efficiency of Pt(1 1 1) in dye-sensitized solar cells confirms the predictions of their theoretical study with the understanding of the mechanism of triiodide reduction at Pt surfaces^9^. The distorted electronic structure in the surface layer also induces exotic physical phenomena in the conductivity and magnetic coupling. The topological insulator is one such example of unique surface behavior^10^. The electronic band structure in the bulk of a non-interacting topological insulator resembles that of a normal insulator with the Fermi level falling in the gap of the conduction and valence bands. While the surface of a topological insulator shows special states falling within the bulk energy gap and allowing surface metallic conductive behavior. Lu *et al.* demonstrated the influence of facet effect on electrochemical performance of one-dimensional SnS nanobelts grown along the [0 2 0] direction and expose (1 0 0) facets^11^. The SnS nanobelts also showed unexpected strong photon absorption properties from the ultraviolet to the near-infrared region.

It is expected that facet engineering might be a possible way to modulate magnetism, because the magnetism is determined by the short-range interaction between the magnetic ions. The short-range interaction depends on the bond length, bond angle, and coordination environment of the magnetic ions. Facet engineering can tune these parameters through surface reconstruction to control the magnetic behavior. Indeed, Ohnishi et al demonstrated theoretically that in iron the magnetic moment increases from 0.73 *μ*_B_/atom in the center layer to 2.98 *μ*_B_/atom in the surface layer of an Fe (0 0 1) plane^12^. For the transition metal oxides, such as manganese oxides, the bond angle of Mn-O-Mn is 180° in the MnO_6_ octahedral environment. The Mn ions are antiferromagnetically coupled through the O superexchange interaction. On the other hand, the bond angle of Mn-O-Mn is 90° in the MnO_4_ tetrahedral environment. Mn-Mn can then show ferromagnetic coupling behavior through Heisenberg exchange coupling^13^. Our previous work indicates that *α*-MnO_2_ nanowires exposed (2 1 0) planes on the side walls show extrinsic spin-glass performance with exchange-bias behaviour^14^.

This work demonstrates that the magnetism and electrochemical properties of *α*-MnO_2_ nanowires can be modulated by exposing different crystal planes on the surface. We synthesized two batches of *α*-MnO_2_-based nanowires, one with exposed (1 1 0) planes on the side walls (defined as MnO_2_-110) and the other with exposed (2 1 0) planes on the side walls (defined as MnO_2_-210). It is interesting that the exposed surfaces of MnO_2_ show significant influences on the magnetic and electrochemical properties of the materials. Magnetic measurements clearly demonstrate that MnO_2_-110 is ferromagnetic (FM) and MnO_2_-210 is mainly antiferromagnetic (AFM). Density functional theory (DFT) calculations confirm the different types of surface magnetism in these two samples. Collectively, we demonstrate two distinct sources contributing to the magnetism in the nanostructures: antiferromagnetic ordering in the core region and tuneable surface magnetism, which is mainly attributed to the surface Mn ions. It is also demonstrated that different exposed surfaces endow unique photocatalyst and lithium battery applications of *α*-MnO_2_ nanowires. The energy-related applications of *α*-MnO_2_ nanowires have been studied by taking advantage of the big size of the (2 × 2) tunnels along the *c*-axe as ion/molecule channels^15^,^16^,^17^,^18^,^19^,^20^,^21^, but the other possibility of their use as magnetic nanowires (MNWs) has been largely unexplored. Inspired by the intriguing structure of *α*-MnO_2_, naively, one may envisage that by selective cutting of the (2 × 2) tunnels along the planes with low Miller indices, such as (1 1 0) and (2 1 0)^22^,^23^, the tunnel structure of *α*-MnO_2_ may be opened up, and consequently, different magnetic and electrochemical performances could be obtained. This reveals a possible route towards the selective modulation of the magnetic and chemical properties in nanostructured *α*-MnO_2_.

## Results and Discussion

### Phase, microstructure, and valence state

**Figure 1:**
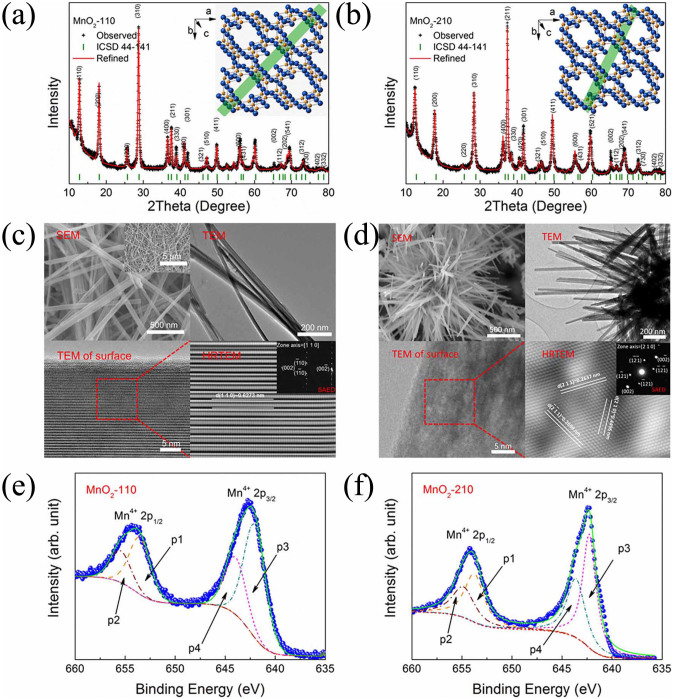
Phase, microstructure, and valence state. (a), Indexed XRD pattern with refinement results of MnO_2_-110 (refined with Rietica, weighted profile R-factor, R_wp_: 13.50). (b), Indexed XRD pattern of MnO_2_-210 (refined with Rietica, R_wp_: 12.80). The insets show the square tunnel structure of *α*-MnO_2_ with space group *I*4/*m*. The (1 1 0) and (2 1 0) planes are highlighted by the green shaded areas, respectively. (c), Microstructural observation results for MnO_2_-110: SEM image with inset to show the ultra-long nature of the nanowires, TEM image, TEM image of surface for a single nanowire, and HRTEM image of surface for a single nanowire with inset SAED pattern. (d), Microstructural observation results for MnO_2_-210: SEM image, TEM image, TEM image of surface for a single nanowire, and HRTEM image of surface for a single nanowire with inset SAED pattern. The HRTEM images of the surface and the SAED patterns (c), (d) indicate that the *α*-MnO_2_ nanowires have exposed (1 1 0) planes and (2 1 0) planes, respectively. (e), (f), X-ray photoelectron spectra of Mn 2p in *α*-MnO_2_ nanowires: MnO_2_-110 (e) and MnO_2_-210 (f). Fitted peaks p1 and p2 are responsible for the observed 2p_1/2_ peak of Mn^4+^, and fitted peaks p3 and p4 for the 2p_3/2_ peak.

Both samples are high purity *α*-MnO_2_, as indicated by the X-ray diffraction (XRD) patterns shown in Figure 1(a, b). All peaks were indexed by *α*-MnO_2_ (ICSD: 44–141). The refined lattice constants are *a* = *b* = 0.9840 nm, *c* = 0.2856 nm for MnO_2_-110, and *a* = *b* = 0.9871 nm, *c* = 0.2845 nm for MnO_2_-210, respectively. Compared with the reported lattice parameters of *α*-MnO_2_, *a* = *b* = 0.9785 nm and *c* = 0.2863 nm (ICSD: 44–141), the lattice expansion in the [*h k* 0] directions is due to the loosened lattice constraints in the nanostructures. On the other hand, the [0 0 *l*] constant decreases slightly. These two samples have obvious differences in preferred growth orientation, as judged from the intensity of the diffraction peaks. The refinement also indicates slight orientations along the [3 0 0] and [1 0 0] directions in both samples. The orientation also comes from the high aspect ratio of their nanowire structures. The nanowires lie on the substrates during XRD measurements. All samples exhibit sharp diffraction peaks, indicating their highly crystalline nature, which is consistent with the high resolution transmission electron microscope (HRTEM) observations.

Microstructures of the MnO_2_-110 were observed by scanning electron microscopy (SEM) and by transmission electron microscopy (TEM), as shown in Figure 1(c). The morphology of the sample with (1 1 0) planes exposed consists of ultra-long nanowires with width of 30 nm and length of more than 10 *μ*m, as shown in the inset of the SEM image. The HRTEM images and the selected area electron diffraction (SAED) pattern indicate that the *α*-MnO_2_ nanowires have (1 1 0) planes exposed on the side walls, which is in agreement with the XRD refinement results. MnO_2_-210 has nanowires with a rectangular morphology, with width of ~20 nm and length of ~1 *μ*m, based on SEM and TEM observations (Figure 1(d)). The HRTEM images and the SAED pattern indicate that the *α*-MnO_2_ nanowires have (2 1 0) planes exposed on the side walls, which is in agreement with the XRD refinement results. The HRTEM images of the surface of a single nanowire reveal the highly crystalline nature of the *α*-MnO_2_ nanowires. The different growth speeds of the different planes and the electronic structure are responsible for this structural variation. It should be noted that the tetragonal crystal structure (with space group I 4/m) of *α*-MnO_2_ shows different preferred growth directions in different reaction environments.

The surface sensitive X-ray photoelectron spectroscopy (XPS) technique was employed to examine the valence state of Mn ions in *α*-MnO_2_ nanowires. The survey scan indicates both MnO_2_-110 and MnO_2_-210 are high impurity samples, as shown in Figure S1. The resulting high resolution scans of Mn-2p_1/2_ and Mn-2p_3/2_ were fitted with four Gaussian-Lorentz peaks, p1–p4, respectively, as shown in Figure 1(e, f), where p1 and p2 are responsible for the observed 2p_1/2_ peak of Mn^4+^, and p3 and p4 for the 2p_3/2_ peak. For MnO_2_-110, the binding energies of p1 and p3 are 653.78 and 642.41 eV, respectively, which can be attributed to the loose surface structure around the MnO_6_ octahedra. The binding energies of p2 and p4 are 654.65 and 642.76 eV, respectively, which are attributed to the body MnO_6_ octahedra. These two different states are present in the ratio of ~1.9:1 in the detected depth of the sample. Similarly, for MnO_2_-210, the binding energies of p1 and p3 are 653.75 and 642.26 eV, while for p2 and p4, they are 654.85 and 643.60 eV, respectively. The two different states are present in the ratio of ~1.4:1 in the detectable depth of XPS. Thus, it is concluded that the amount of surface MnO_6_ octahedra in MnO_2_-110 is higher than in MnO_2_-210 due to the rougher surface. The lower oxidization states of Mn, such as Mn^3+^ and Mn^2+^, were not detected, or their contents were below the detectable limits of XPS, in both MnO_2_-110 and MnO_2_-210.

### Magnetic properties

**Figure 2:**
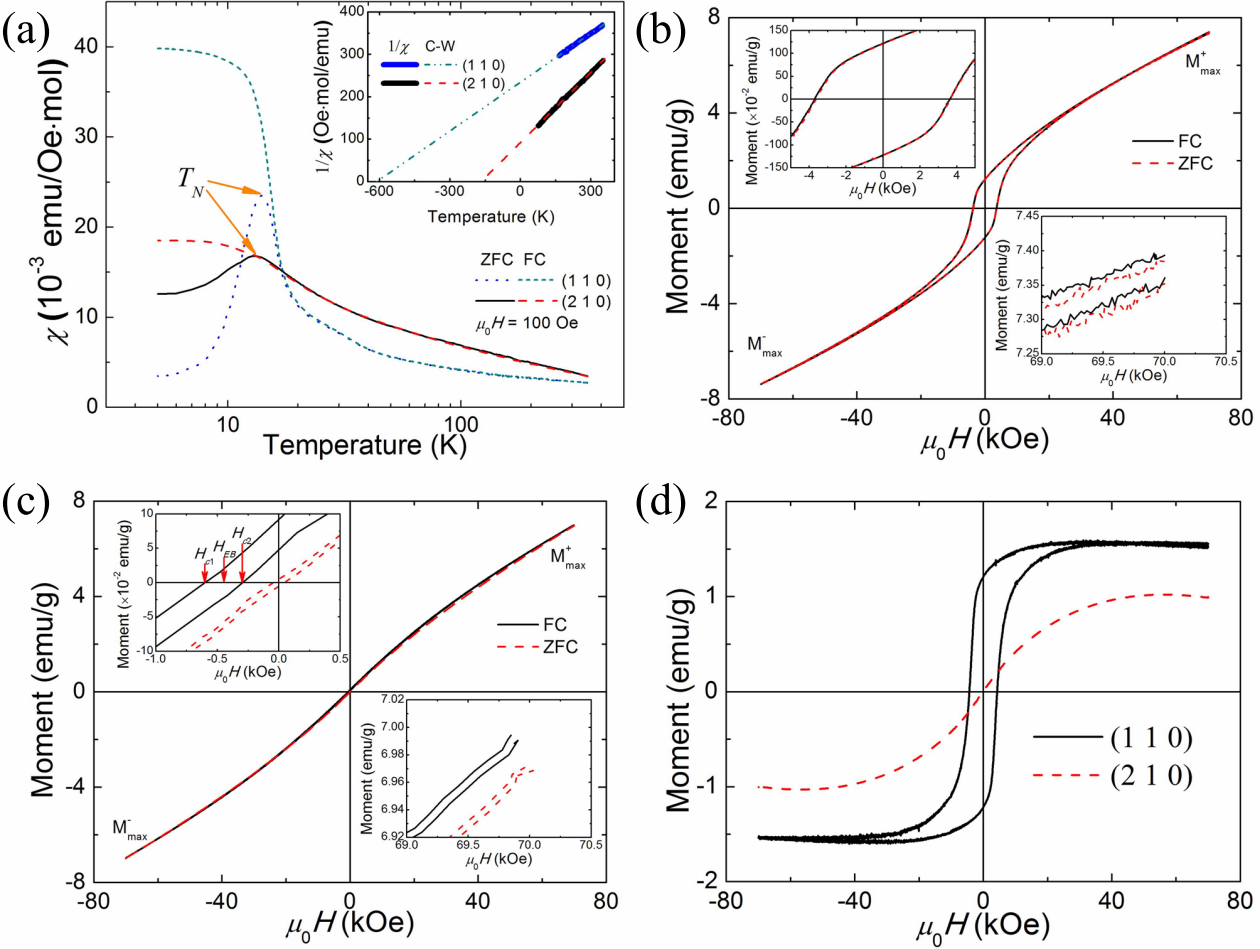
Magnetic properties. (a), Magnetic behavior of *α-*MnO_2_ nanowires: *χ*(*T*) *vs. T* curves after zero field cooling (ZFC) and after field cooling (FC). The inset shows the ZFC 1/*χ*(*T*) *vs. T* curve fitted by the Curie-Weiss law: 1/*χ*(*T*) = (*T*−*θ*)/*C*, as indicated by the dot-dashed line. (b), (c), Hysteresis loops measured at 5 K after ZFC or FC for MnO_2_-110 (b) and MnO_2_-210 (c). 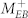 is the positive maximum magnetization and 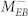 is the negative maximum magnetization. The upper left insets show the high residual magnetism and strong coercive fields, with the left and right coercive fields *H_C_*_1_ and *H_C_*_2_, and the exchange bias field *H_EB_* marked in (c). The lower right insets show the open loops due to magnetization relaxation. (d), Comparison of surface magnetic moments.

*α*-MnO_2_ has been reported as an antiferromagnetic substance with a Néel temperature (*T_N_*) of ~24.5 K^24^. Both zero-field-cooled (ZFC) and field-cooled (FC) susceptibility were measured under a 100 Oe magnetic field, and the results are shown in Figure 2(a). The ZFC curve bifurcates from the FC one below ~13 K and shows a peak at ~13 K for both samples. The bifurcation indicates that the magnetic phase is making a transition from paramagnetism (PM) to a spin-glass-like state in the *α*-MnO_2_ nanowires^25^, which is similar to the previously reported transition temperature in *α*-MnO_2_ nanowires^26^. The spin-glass moments are easily polarized under low magnetic field, while the AFM susceptibilities are much lower. This means that that the characteristic of the AFM transition is almost buried in the ferromagnetic cluster ordering. Careful observation can also find the weak AFM transition feature in MnO_2_-110 between 20 and 30 K. The high temperature susceptibility data for *α*-MnO_2_ are in good agreement with the Curie–Weiss law and therefore can be fitted to the equation 1/*χ*(*T*) = (*T*-*θ*)/*C*, where *θ* is the Curie–Weiss temperature, and *C* is the Curie–Weiss constant. The fitted result is presented in the inset of Figure 2(a). The 1/*χ*(*T*) of MnO_2_-110 was fitted with antiferromagnetic part with *θ* = −621 K and *C* = 2.608 emu·K/Oe·mol with contribution of 98.3% and ferromagnetic part with *θ* = −33.7 K and *C* = 5.216 emu·K/Oe·mol with contribution of 1.7%. The parameters are *θ* = −166 K and *C* = 1.816 emu·K/Oe·mol for MnO_2_-210, respectively. The negative *θ* value indicates the antiferromagnetic behaviour of the *α*-MnO_2_ nanowires. The *θ* value of MnO_2_-110 is much lower than that of MnO_2_-210, implying a much stronger antiferromagnetic coupling in MnO_2_-110. The susceptibility values of MnO_2_-210 increase gradually with cooling temperature and do not show sudden transition at *T_N_*. In contrast, the transition of MnO_2_-110 shows intensive susceptibility variation and its absolute susceptibility values are lower than those of MnO_2_-210 when the temperature is higher than ~20 K. Furthermore, the temperature dependence of susceptibility is quite weak compared with that of MnO_2_ in the high temperature region. Interestingly, the *T_N_* values in the two samples are similar. This can be understood that the coupling of the majority of the inside atoms in both samples is antiferromagnetic - resembling the case in bulk MnO_2_. Nevertheless, the distinct surface magnetic ground states of (2 1 0) and (1 1 0) show great influences on the Curie-Weiss Temperatures.

The 5 K hysteresis loops of MnO_2_-110 after ZFC or FC under 10 kOe magnetic field from 350 down to 5 K are presented in Figure 2(b), with measurements between ±70 kOe. Both the ZFC and the FC loops deviate from antiferromagnetism under magnetic field, showing high remnant magnetism and a strong coercive field, as shown in the upper left inset of Figure 2(b). This provides evidence of a mixed state of a component from the antiferromagnetic core of MnO_2_ combined with stable net surface spins. The high remnant magnetism indicates the great amount of net magnetic spin on the surface, and the strong coercive field indicates anisotropic magnetic coupling. The hysteresis loops are not saturated under ±70 kOe due to the contribution of the antiferromagnetic core as well as the spin-glass component, which is a common phenomenon in the case of nanocrystalline compounds, alloys, and oxide materials^27^,^28^. The open loop, as shown in the lower right inset of Figure 2(b), is a characteristic of spin-glass^27^, with slow dynamics. The positive maximum magnetization 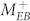 and the negative maximum magnetization 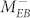 under ±70 kOe show symmetric behaviour with a very small difference below 0.019 emu/g, which is defined as the magnetization exchange bias: 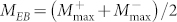. Exchange bias behaviour is a quite normal phenomenon in nanoparticles with size/surface induced ferromagnetic clusters. The magnitude of the exchange bias effect is usually compared quantitatively, using the following two fields, the exchange bias field, *H_EB_*, and the coercive field, *H_C_*, defined as *H_C_* = |*H_C_*_1_ - *H_C_*_2_|/2 and *H_EB_* = (*H_C_*_1_ + *H_C_*_2_)/2, where *H_C_*_1_ and *H_C_*_2_ are the left and right coercive fields, respectively. The *H_C_* of MnO_2_-110 approaches 3667 Oe, while the *H_EB_* is only ~−50 Oe.

Figure 2(c) shows the hysteresis loops of MnO_2_-210. The loops deviate slightly from linear behaviour. Both the remnant magnetism and the coercive field are quite weak compared with those of MnO_2_-110, as shown in the upper left inset of Figure 2(c). The open loop phenomenon was also observed, as shown in the lower right inset of Figure 2(c). Obvious exchange bias behaviour was observed in the FC loop, but is absent from the ZFC loop. *H*_max_, the maximum applied magnetic field, is crucial for investigating the exchange bias effect, because small *H*_max_ may lead to the displacement of the magnetic hysteresis loop, even for FM and glassy magnetic substances. This is attributed to the irreversible magnetization processes known as minor loop effects^29^. When *H*_max_ is small, the FC hysteresis loops are always shifted towards the negative field and positive magnetization. The *M_EB_* value is very small (less than 0.018 emu/g) in the *M*(*H*) loop. Thus, the exchange bias effect is indeed present in the *α*-MnO_2_ nanowires. The *H_C_* of MnO_2_-110 is ~154 Oe, and the *H_EB_* is ~−451 Oe. The exchange bias effect is much stronger in MnO_2_-210 than in MnO_2_-110.

Comparing the magnetization behaviour of MnO_2_-110 and MnO_2_-210, it is found that the former sample shows much stronger and more stable net magnetic coupling, as shown in Figure 2(d), but does not display strong exchange bias behaviour. The difference is attributed to their surface structures: the (1 1 0) plane contains chains of MnO_6_ octahedra on the smooth matrix of (2 × 2) tunnels, while the (2 1 0) plane forms a step-type surface with chains of MnO_6_ octahedra. The higher density of MnO_6_ octahedral chains in MnO_2_-110 is responsible for its strong magnetization. On the other hand, the weak coupling between the core AFM spins and the surface spins cannot generate an intensive exchange bias when the magnetic field is reversed. In MnO_2_-210, the core AFM spins couple intensively to the weak surface spins during the magnetic field reversal, which is the origin of the exchange bias behavior.

**Figure 3:**
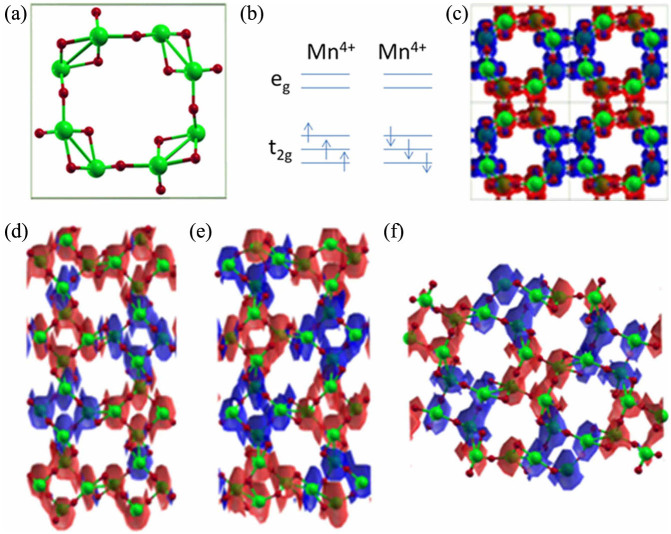
Atomic structure and ground magnetic state of bulk *α*-MnO_2_ and different surfaces. (a), top view of the unit cell with 24 atoms, a (2 × 2) tunnel. (b), nearest neighbour Mn^4+^-Mn^4+^ pair electron configuration and magnetic alignment. (c), the resulting (calculated) magnetic structure showing the isosurface plots of the spin density with the isosurface value being 0.02 electron/Å^3^. The isosurface plots of the spin density of (d), O-terminated (1 1 0) surface. (e), Mn-terminated (1 1 0) surface. (f), (2 1 0) surface, with the isosurface value being 0.02 electron/Å^3^. Note the (2 × 2) tunnels in the bulk sample (c) and exposed (2 1 0) plane (f) have the same anti-parallel spin alignment, while parallel alignment occur in the exposed (1 1 0) planes (as shown in (d) and (e)). Big (green) balls represent Mn ions and small (dark red) balls represent O ions. The red and blue spherical shells indicate spin-up and spin-down, respectively. The covered space indicates the intensity of the moments of individual ions.

Owing to their inherent shape anisotropy and the ability to incorporate different components, magnetic nanowires (MNWs) offer unique magnetic properties distinct from those of bulks, thin films, and particles^30^,^31^,^32^,^33^. A key property of MNWs lies in the strong coupling of magnetic properties with the nanowire orientations^34^. For practical applications, it is desirable to synthesize nanowires with tuneable magnetic ordering, as they can offer greater flexibility in the design and optimization of nanodevices. Bulk *α*-MnO_2_ has a Hollandite-type structure (tetragonal; space group I4/m; *a* = 9.777 Å and *c* = 2.855 Å)^35^. This tunnel-structured oxide is characterized by double chains of edge-sharing MnO_6_ octahedra, which are linked at corners to form one-dimensional (1D) (2 × 2) and (1 × 1) tunnels that extend in a direction parallel to the *c*-axis of the tetragonal unit cell (Figure 3(a)). According to Néel's model^36^, neighboring pairs of octahedrally coordinated Mn^4+^ (d^3^, S = 3/2) ions are aligned antiparallel to each other (Figure 3(b)), leading to an antiferromagnetic ground state (see Figure 3(c) and Supplementary Figure S2).

To confirm the influence of the surface on the magnetism, density functional theory (DFT) calculations were performed to distinguish the magnetism of bulk *α*-MnO_2_ from those of exposed (1 1 0) and (2 1 0) surfaces. Bulk *α*-MnO_2_ possesses an antiferromagnetic ordering between the corner-sharing MnO_6_ octahedra and a ferromagnetic ordering between the edge-sharing MnO_6_ octahedra, as shown in Figure 3(c) and Supplementary Figure S2. The corresponding ferromagnetic structure is only 12 meV per cell higher in energy. The magnetic moments of Mn^4+^ and O^2−^ are ± 2.79 *μ*_B_ and ~± 0.1 *μ*_B_, respectively. These values are in excellent agreement with the 3 *μ*_B_ per Mn based on crystal field theory.

We used supercells containing symmetric slabs with inversion symmetry to simulate the MnO_2_-110 and MnO_2_-210 surfaces. For MnO_2_-110, we considered the stoichiometric O-terminated (96 atoms) and non-stoichiometric Mn-terminated (92 atoms) surfaces, denoted as (1 1 0)-O and (1 1 0)-Mn, respectively. For MnO_2_-210, we only considered the stoichiometric cell, containing 96 atoms. For each surface, to obtain the ground-state magnetic structure, various possible magnetic alignments for the surface or subsurface layers have been considered and compared. The calculated spin-density isosurface plots for the bulk (shown in a 2 × 2 × 2 cell) and the plots for (1 1 0)-O, (1 1 0)-Mn, and MnO_2_-(2 1 0) are shown in Figure 3(d–f), respectively. For the (1 1 0)-O, in the ground magnetic state, the Mn surface layer and the first subsurface layer are coupled ferromagnetically (Figure 3(d)). The Mn magnetic moments are 2.94 *μ*_B_ and 2.88 *μ*_B_. For the (1 1 0)-Mn (Figure 3(e)), which involves strong structural distortion of the surface atoms, in the ground state, only the surface Mn layer is coupled ferromagnetically, with the Mn atomic moment being 4.01 *μ*_B_, suggesting Mn^3+^ ions. The Mn moments in the subsurface layer are 2.75 *μ*_B_, close to the moment in the bulk, 2.80 *μ*_B_. Considering the absence of the Mn^3+^ state in the XPS measurements, we tentatively conclude that the MnO_2_-110 samples prepared by hydrothermal reaction (can be regarded as an O-rich condition) are exposed with stoichiometric (1 1 0)-O surfaces. More possible magnetic configurations of oxygen- and manganese-terminated (1 1 0) surfaces are listed in Supplementary Figures S3 and S4 and compared with the ground states. The MnO_2_-210 surface is also characterised by strong distortion, as shown in Figure 3(f). As a result, the Mn spin moment values vary substantially, ranging from 0.8 *μ*_B_ to 3.7 *μ*_B_. Yet, the magnetic structure of the MnO_2_-210 surface does not show any obvious difference compared with the one in the bulk. Its exchange bias behaviour is attributed to the surface-defect-induced magnetic spin coupling with the AFM core. It is important to note that our DFT calculations predict a strong ferromagnetic coupling for both the (1 1 0)-O and the (1 1 0)-Mn surfaces, although there is only a thin depth where ferromagnetism applies. For MnO_2_ (1 1 0)-O, the exchange energy (defined as the energy difference between antiferromagnetic and ferromagnetic interactions) in the outermost surface Mn layer is 298 meV/2Mn, and it rapidly decreases to 39 meV/4Mn in the second Mn layer. For MnO_2_ (1 1 0)-Mn, the exchange energy in the outermost surface layer is 533 meV/2Mn. The observed suboptimal weak ferromagnetism is likely to be due to the rough surfaces. Atomically smooth surface preparation is notoriously challenging – especially for chemical growth methods such as we employed in this study. Nevertheless, such intrinsic strong surface ferromagnetism may pave the way to interesting practical applications. To this end, further progress will require novel manufacturing techniques that allow control over nanowires so as to achieve atomic smoothness.

### Tuneable electronic bandgap energy

**Figure 4:**
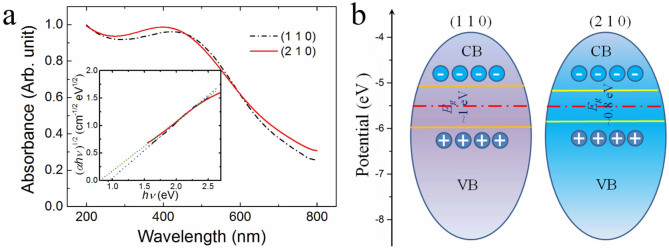
Light absorbance and bandgap properties of MnO_2_-110 and MnO_2_-210. (a), UV-Vis absorption spectra of the *α*-MnO_2_ nanowires. Inset shows the (*αhv*)^1/2^ vs. *hv* plots (*α*, absorption coefficient; *hv*, photon energy). Bandgap values of 0.98 eV and 0.84 eV can be deduced for the exposed (1 1 0) sample and the exposed (2 1 0) sample, respectively. (b), Sketch of possible bandgap alignments of MnO_2_-110 and MnO_2_-210.

MnO_2_ has been demonstrated to be a highly efficient photocatalyst^37^, either alone or in MnO_2_/TiO_2_ heterogeneous photocatalysts^38^,^39^. Figure 4(a) shows the ultraviolet-visible (UV-vis) absorption spectrum of the *α*-MnO_2_ nanowires. Broad absorption bands ranging between 300 and 600 nm with peak positions of ~400 nm for MnO_2_-210 and ~450 nm for MnO_2_-110 are observed. The *d−d* transitions of Mn ions in the *α*-MnO_2_ nanowires is responsible for the absorption in the visible light range. The Mn 3*d* energy level splits into lower (t_2g_) and higher (e_g_) energy levels in the ligand field of MnO_6_ octahedra, and the energy difference between the *e_g_* and *t_2g_* states is responsible for the optical bandgap energy^40^. The bandgap energy *E*_g_ for the *α*-MnO_2_ nanowires was estimated using the Kubelka-Munk function to plot the product of the square root of the absorption coefficient and the photon energy against the incident photon energy (*hv*)^41^. A straight line in a photon energy range close to the absorption threshold can be fitted, as shown in the inset of Figure 4(a). *α*-MnO_2_ nanowires have an indirect electronic transition near the bandgap^41^,^42^. The bandgap energy for the *α*-MnO_2_ nanowires can be derived as 0.98 eV for the sample with the exposed (1 1 0) planes, while it is 0.84 eV for the sample with exposed (2 1 0) planes, as derived from the intercept of the linear portion with the abscissa. Remarkable differences in the optical properties of nanostructured MnO_2_ materials were previously observed. For example, Pereira *et al.* found that the absorption of MnO_2_ colloid at longer wavelengths strongly decreases as the MnO_2_ particles become smaller^43^. Gao *et al.* observed a bandgap of 1.32 eV in *α*-MnO_2_ nanofibers with typical diameters of 20–60 nm and lengths of 1–6 *μ*m^44^. Sakai et al. also reported that MnO_2_ nanosheets with a very small thickness of about 0.5 nm had bandgap energy of about 2.23 eV^41^. The shift in the bandgap to higher energies can be attributed to the carrier confinement in the small semiconductor particles. Figure 4(b) is a sketch of the possible bandgap alignment of MnO_2_. Selective surface engineering can be an effective tool to control the driving force of charge transport and charge separation.

### Electrochemical properties

**Figure 5:**
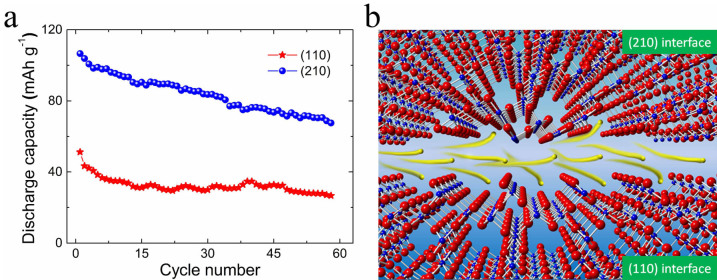
Electrochemical properties of MnO_2_-110 and MnO_2_-210. (a), Comparison of discharge capacity cycling performance of *α*-MnO_2_ nanowires with different exposed crystal planes. The (2 1 0) exposed sample shows much higher lithium battery performance. (b), Schematic diagram of lithium ions (yellow balls) and their intercalation/absorption performance as they interact with exposed (1 1 0) and (2 1 0) surfaces in electrolyte.

Lithium storage properties of the *α*-MnO_2_ nanowires were investigated using the galvanostatic charge–discharge method. The capacity difference between the two samples is obvious, as shown in Figure 5(a). The origin of the performance variation in *α*-MnO_2_ nanowires can be attributed to the different intercalation/absorption behavior of lithium ions as they interact with exposed (1 1 0) and (2 1 0) surfaces, as illustrated in Figure 5(b). The capacity of the batteries depends on the intercalation Li^+^ ions in *α*-MnO_2_ lattice under the charge/discharge voltage. One of the determinant factors is the penetration ability of Li^+^ ions in the electrolyte through the close-packed plane of MnO_6_ into the (2 × 2) tunnels. The more the (2 × 2) tunnels exposed to electrolyte, the more chance for the Li^+^ ions intercalate into the *α*-MnO_2_ lattice. Judging from the theoretical period structure of outmost layers of exposed with (1 1 0) and (2 1 0) plane as demonstrated in Figure S5, the (2 × 2) tunnels have more chance to accept Li^+^ ions to build up the capacity of the material. The MnO_6_ as blockers on the (1 1 0) and (2 1 0) surfaces were highlighted in Figure S5 to demonstrate the intercalation chance for Li^+^ into the (2 × 2) tunnels. It can be roughly estimated that direct exposure rate of the (2 × 2) tunnels to electrolyte is 2/3 when the (1 1 0) plane was the exposed facet. The rate increased to 4/5 for (2 1 0) plane as the exposed facet. This may be one of the reasons that the capacity of MnO_2_-210 is double to that of MnO_2_-110.

In summary, the evidences of facet of nano particles suggest the facet control of nano materials for practical application is essential for the exploration of high performance. The magnetic property dependence on exposed crystal plane of *α*-MnO_2_ nanowires reveals that the variation of the size and morphology dependence of nanomagnetism and electrochemical reaction should be examined to explain the origin of the performance difference and maximize the performance through facet control engineering.

## Methods

### Synthesis of MnO_2_-110

To synthesize *α*-MnO_2_ nanowires with different exposed planes, two different hydrothermal reaction processes were employed in Teflon-lined autoclaves. Rectangular MnO_2_-110 was synthesized by a hydrothermal method with procedures reported in Wang et al.'s^45^ and Gao et al.'s work^46^. KMnO_4_ (Aldrich, 99.0%) and NH_4_F (Aldrich, 99.99%) were used to form *α*-MnO_2_ under neutral hydrothermal conditions. In a typical procedure, KMnO_4_ (0.001 mol) and NH_4_F (0.001 mol) were dissolved under magnetic stirring in 40 mL doubly deionized water to form a clear solution. The solution was transferred into a 50 mL autoclave with a Teflon liner. The autoclave was sealed and maintained at 150°C for 24 h, and then cooled to room temperature naturally. The suspension was then alternately centrifuged with doubly deionized water and ethanol several times, and the resulting brown precipitate was dried in an oven at 80°C for 10 h.

### Synthesis of MnO_2_-210

Rectangular MnO_2_-210 was synthesized with a Mn^2+^ source: Mn^2+^ + (NH_4_)_2_S_2_O_8_ + 2H_2_O → MnO_2_ + (NH_4_)_2_SO_4_ + 2H_2_SO_4_. H_2_SO_4_ was added to the solution to adjust its pH value, since the size and morphology of the nanostructures show a strong dependence on the pH value of the formation environment^21^. In a typical synthesis, MnCO_3_ (Aldrich, 99.9%), (NH_4_)_2_S_2_O_8_ (Aldrich, >98%), HNO_3_ (>90%), and H_2_SO_4_ (Aldrich, 95–98%) were used as received without further purification. MnCO_3_ (0.02 mol) was dispersed in deionized water (200 mL), and HNO_3_ (0.04 mol) was then added to make a transparent solution. Then, (NH_4_)_2_S_2_O_8_ (0.02 mol) was added, and the solution was diluted to 300 mL. After the addition was completely dissolved, concentrated H_2_SO_4_ (20 mL) was added, and the solution was diluted to 400 mL and stirred for 30 min. The hydrothermal treatment was performed in a Teflon-lined autoclave, with heating at 140°C for 1 hour in a microwave device. After the reaction was completed, the solution was cooled to room temperature, and the resulting suspension was centrifuged in order to separate the precipitate from the supernatant liquid. The precipitate was washed and centrifuged two times and then dried at 80°C overnight.

### Physical characterization

Both samples were microstructurally characterized by X-ray diffraction (XRD: GBCMMA, Cu K*_α_*, *λ* = 0.154056 nm) in conjunction with Rietveld refinement (Rietica), UV-Visible spectrophotometer (Cary 5000 UV-Vis-NIR, Agilent), X-ray photoelectron spectroscopy (XPS: EscaLab 220-IXL, Al K*_α_*), field emission gun scanning electron microscopy (FEG-SEM: JSM-6700F), and transmission electron microscopy (TEM: JEOL-2010) with high resolution TEM (HRTEM), operating at 200 kV. Selected area electron diffraction (SAED) patterns were also collected for crystal structure analysis. Magnetic properties were measured using a commercial vibrating sample magnetometer (VSM) model magnetic properties measurement system (MPMS: Quantum Design, 14 T) in applied magnetic fields up to 70 kOe. The nanoparticles were filled into a polypropylene powder holder, which is an injection moulded plastic part as powder container during the VSM measurement process. The polypropylene powder holder was mounted into a brass trough, which is made from cartridge brass tubing with a cobalt-hardened gold plating finish. Both polypropylene powder holder and brass trough were made by Quantum Design as commercial VSM sample holders with very low magnetic moments, which are much lower than the moments of *α*-MnO_2_ samples.

### Electrochemical characterisation

Electrochemical characterisation of MnO_2_-110 and MnO_2_-210 was conducted in 2032-type coin cells. The working electrodes were prepared by mixing 80 wt% *α*-MnO_2_ nanowires and 10 wt% carbon black, along with 10 wt% polyvinylidene difluoride (PVdF), in the presence of N-methyl pyrrolidinone (NMP), and this slurry was pasted on aluminium foil and then heat-treated at 80°C under vacuum overnight. CR2032 coin type cells were employed in the battery testing, with lithium foil serving as counter electrode and a porous Celgard polypropylene membrane as separator. The electrolyte consisted of a solution of 1 M LiPF_6_ dissolved in a mixture of the solvents ethylene carbonate and dimethyl carbonate in a volume ratio of 1:1. Galvanostatic charge–discharge measurements were performed over the potential range from 2 V to 4.5 V (vs. Li/Li^+^) at a constant current density of 20 mA/g on a Land CT2001A battery tester.

### First principles simulation

All calculations were performed using spin-polarised DFT with the generalized gradient approximation^47^ (GGA) for the exchange-correlation functional, as implemented in the all-electron *DMol^3^* code^48^,^49^. Earlier study shows that GGA functional predict the correct ground magnetic states for a range of Manganese-oxides^50^. The wave functions are expanded in terms of a double numerical quality localized basis set with a real-space cut-off of 10 bohr. For bulk calculations, the Brillouin zone (BZ) integration was performed using Monkhorst-Pack grids of 12 × 12 × 36 was used. The calculated antiferromagnetic *α*-MnO_2_ lattice constants are *a* = *b* = 9.731 Å and *c* = 2.854 Å, which compare well with the experimental ones. For surface supercells, a 30–40 Å vacuum region is used between adjacent slabs. All surfaces are fully relaxed, while keeping the innermost three centre layers fixed at the bulk values. The Brillouin zone (BZ) integration was performed using Monkhorst-Pack grids of 8 × 8 × 1, with 18 k points in the irreducible part of the BZ for all the surfaces. The convergence criteria for the forces on the atoms are less than 0.01 eV/Å, and for the total energy 0.05 meV.

## Author Contributions

W.X.L. designed the study, with advice from S.X.D. and Z.Q.S. The initial synthesis was performed by W.X.L., R.Z. and W.X.L. obtained the X-ray diffraction data, and microstructural observation and electron diffraction patterns were obtained by W.X.L. and Z.Q.S. Rietveld refinements were initially performed by W.X.L. and XPS were measured and analyzed by Z.Q.S. Magnetic susceptibility was measured and analyzed by R.Z. and W.X.L. The semiconducting properties and lithium battery performances were measured by G.D.D. The density functional theory calculations were performed by X.Y.C. All authors discussed the results; W.X.L. and Z.Q.S. wrote and revised the manuscript, with discussions mainly with X.Y.C., R.K.Z., S.X.D. and S.P.R.

## Supplementary Material

Supplementary Information Supporting Information
