# Supplementary material for: Performance modulation of α-MnO2 nanowires by crystal facet engineering
Source: Sci Rep. 2015 Mar 11;5:8987. doi: 10.1038/srep08987 (PMC4355743; doi:10.1038/srep08987)
Supplement: Supplementary Information — Supporting Information [file srep08987-s1.doc]

Performance modulation of *α*-MnO_2_ nanowires by crystal facet engineering

W. X. Li,^1,2,3,🟉^ X. Y. Cui,^4,5^ R. Zeng,^1^ G. D. Du,^1^ Z. Q. Sun,^1,**^ R. K. Zheng,^4,6^ S. P. Ringer,^4,5^ and S. X. Dou^1^

^1^Institute for Superconducting and Electronic Materials, University of Wollongong, NSW 2522, Australia

^2^School of Materials Science and Engineering, Shanghai University, Shanghai 200072, PR China

^3^Solar Energy Technologies, School of Computing, Engineering and Mathematics, University of Western Sydney, Penrith, NSW 2751, Australia

^4^Australian Centre for Microscopy and Microanalysis, The University of Sydney, NSW 2006, Australia

^5^School of Aerospace, Mechanical and Mechatronic Engineering, The University of Sydney, NSW 2006, Australia

^6^School of Physics, The University of Sydney, Sydney, NSW 2006, Australia

^🟉^e-mail: w.li@uws.edu.au

*^**^*e-mail: ziqi@uow.edu.au

**S1. Survey XPS patterns of MnO_2_-110 and MnO_2_-210.** Oxygen, magnese, and carbon were detected in both samples. The carbon was used to calibrate the XPS peaks shift.


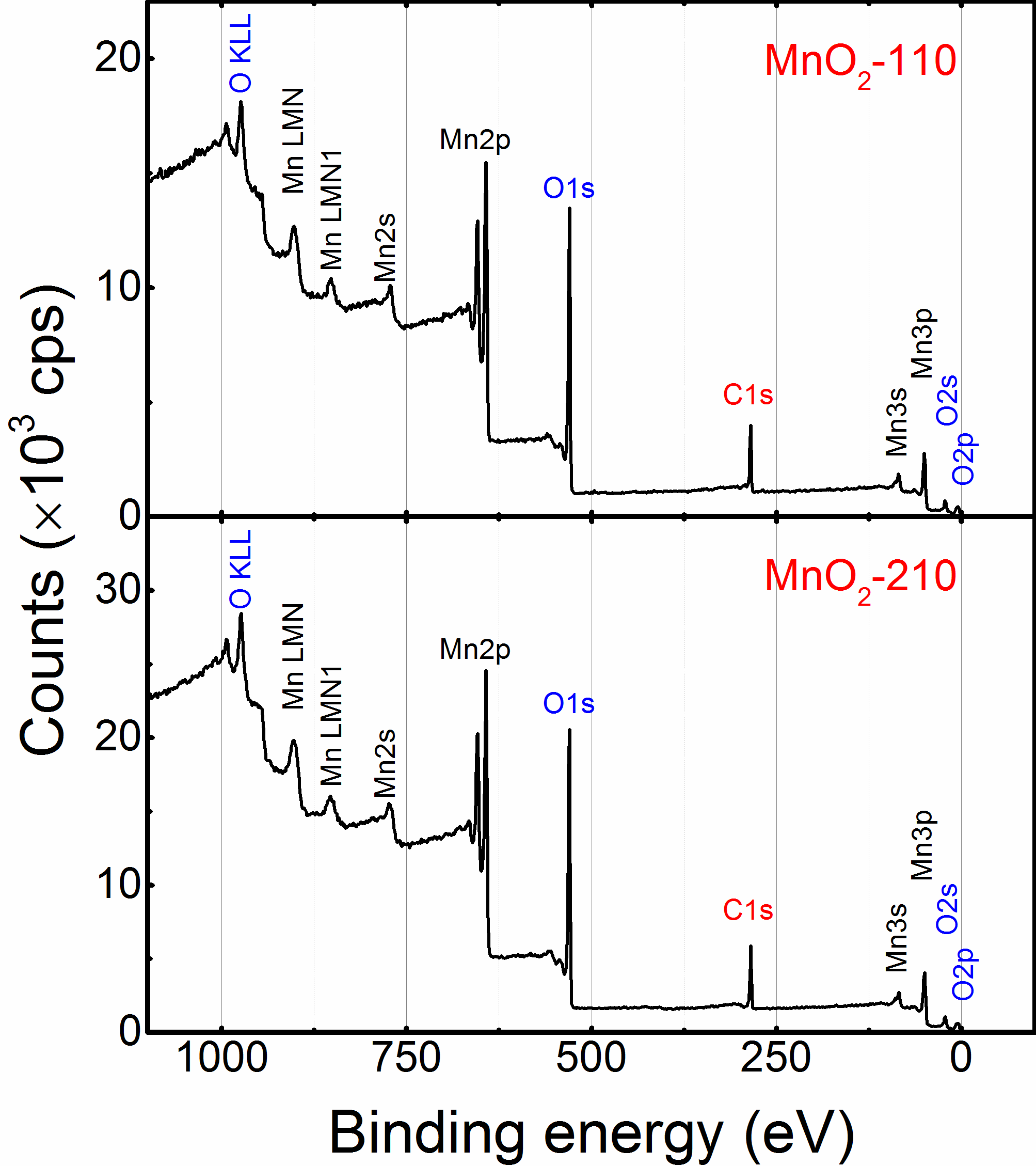


**Figure S1** Survey XPS patterns of MnO_2_-110 and MnO_2_-210.

**S2. Magnetic ground state of bulk MnO_2_.** The DFT predicted magnetic ground state is shown in Figure S2. For bulk MnO_2_, the energy difference between the ferromagnetic state and the antiferromagnetic state is only 11 meV per cell (Z = 8) from the DFT-generalized gradient approximation (GGA). These extremely competitive magnetic phases are believed to facilitate the selective magnetic modulation of the surface.


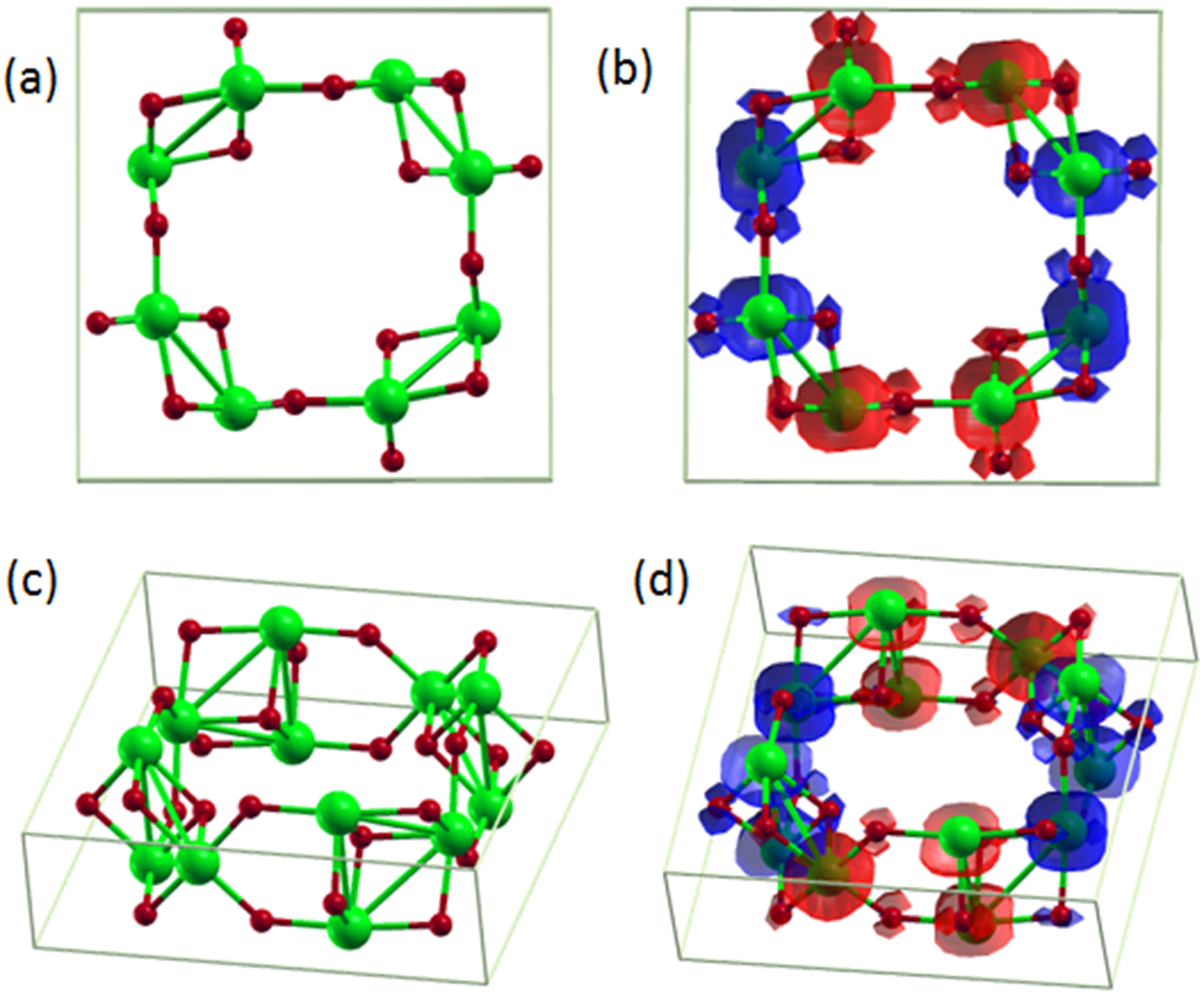


**Figure S2** (a, b) Top view of the unit cell with 24 atoms: (a) (2 × 2) tunnel, and (b) the calculated magnetic structure of bulk MnO_2_. (c, d) Tilted view of unit cell in a 3D space and the magnetic configuration of bulk MnO_2_. Big (green) balls represent Mn ions and small (dark red) balls represent O ions. Red and blue isosurfaces denote spin-up and spin-down, respectively. The absolute total energy of the ground antiferromagnetic state is -10462.422589 Ha.

**S3. Magnetic structures of oxygen-terminated (110) MnO_2_ surface determined by DFT calculations:** This is a stoichiometric surface, and the surface layer does not show obvious distortion compared with the lattice in the bulk. Three possible magnetic configurations were calculated with optimized surface structures, as shown in Figure S3(b-d). The total energy was reduced dramatically in all three models compared with the model without the non-magnetic state. In Figure S3(b), the spin directions of all Mn ions in the body are parallel in a (1 🞨 1) tunnel and then antiparallel with the adjacent tunnels. This is the same as in the bulk model. The total energy becomes significantly lower (by ~ 0.3 eV), however, when there are asymmetric spins in the (1 🞨 1) tunnels on the surface, as shown in Figure S3(c, d). The model with the lowest energy was defined as the ground state, the total energy of which is defined as 0 eV. The energies indicated for the other models were defined by the respective differences from the ground state.


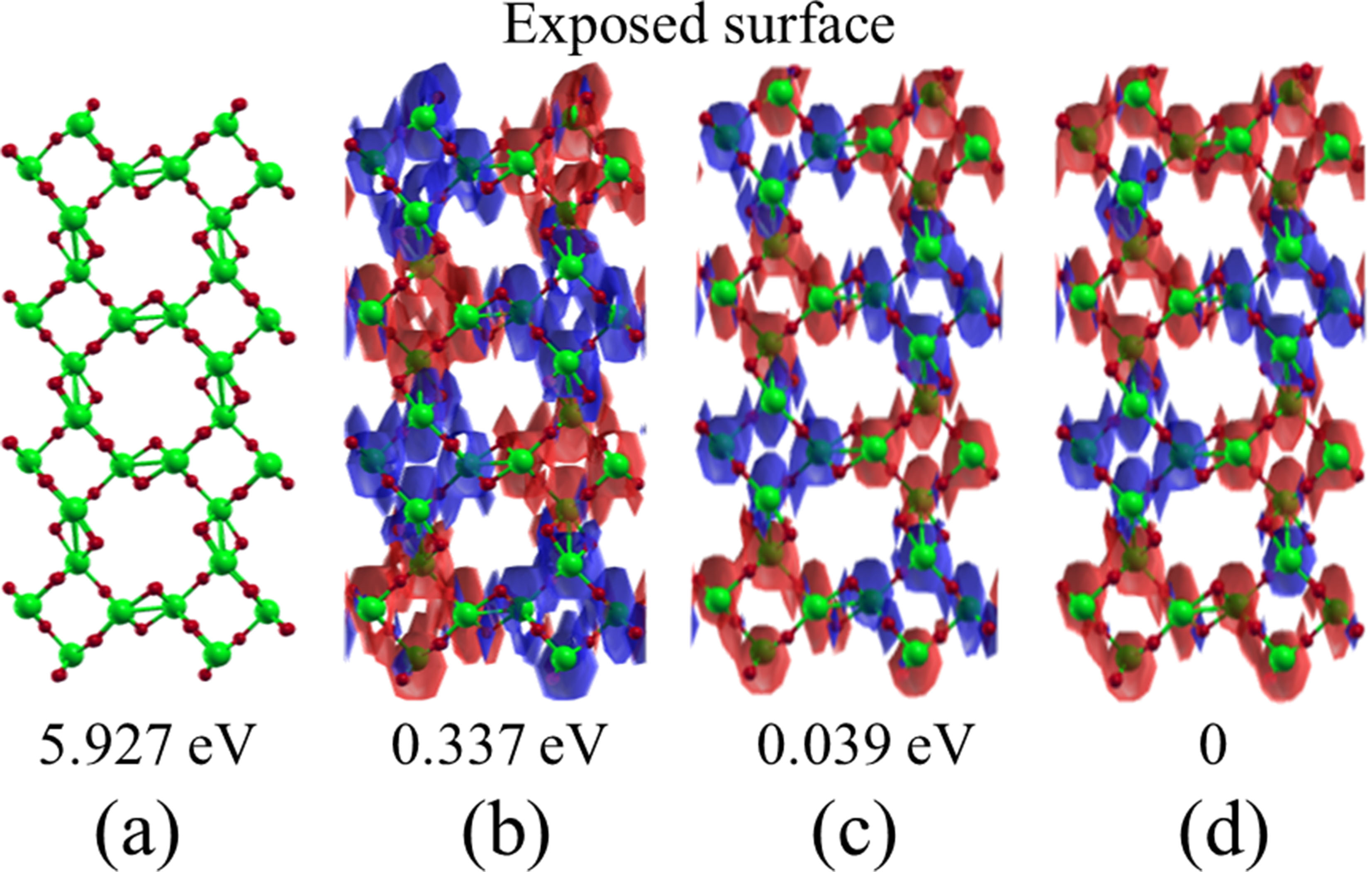


**Figure S3** Calculated magnetic structures and their relative total energies of the oxygen terminated MnO_2_-110 surface: (a) non-magnetic phase and (b-d) three possible magnetic phases. The ground state corresponds to two Mn surface layers coupled ferromagnetically, leading to net magnetic moments in some (1 🞨 1) tunnels on the surface. The absolute total energy of the ground magnetic state is -41849.560834 Ha.

**S4. Magnetic structures of magnesium-terminated (110) MnO_2_ surface determined by DFT calculations:** The non-stoichiometric manganese-terminated surface structure was also simulated, as shown in Figure S4(a). Strong lattice distortion can be observed in the surface (1 🞨 1) tunnel and subsurface (2 🞨 2) tunnel. The valence states of the surface Mn ions vary from Mn^4+^ to lower oxidation states, as confirmed by Mulliken population analysis: the outermost surface Mn ions have a valence of 0.92 eV compared with 1.29 eV in the body. The ground state magnetic configuration also exhibits obvious net magnetic moments, as shown in Figure S4(d). Compared with the total energy of the ground state, the other two magnetic configurations shown in Figure S4(b, c) display much higher total energies.


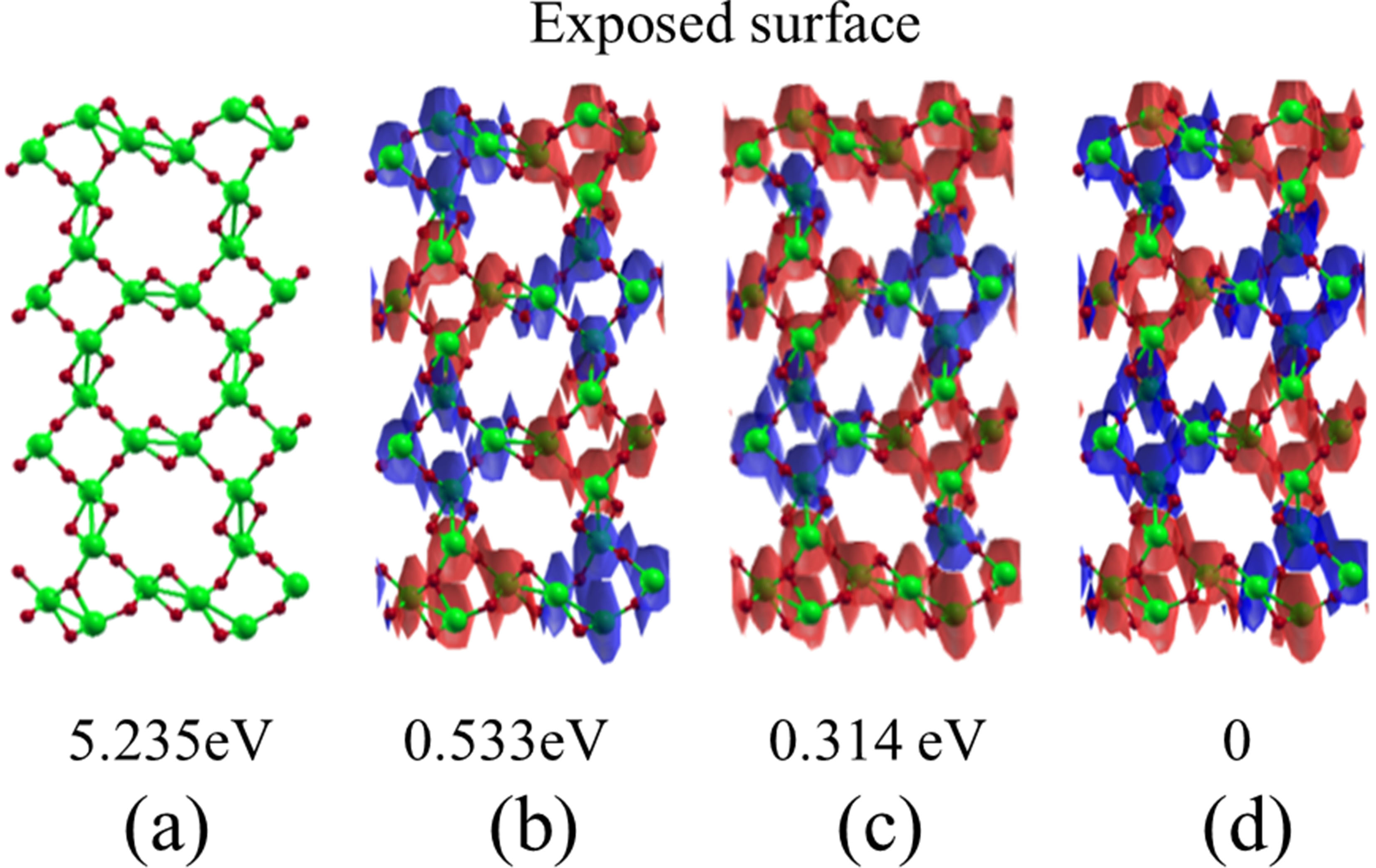


**Figure S4** Calculated magnetic structures and their relative total energies of the magnesium-terminated MnO_2_-110 surface: (a) non-magnetic phase and (b-d) three possible magnetic phases. The ground state corresponds to an outermost Mn surface layer that is coupled ferromagnetically, leading to net magnetic moments in some (1 🞨 1) tunnels on the surface. The absolute total energy of the ground magnetic state is -41598.727095 Ha.

**S5. The crystal structure of the outmost layers for α-MnO_2_ with exposed (1 1 0) and (2 1 0) plane.**


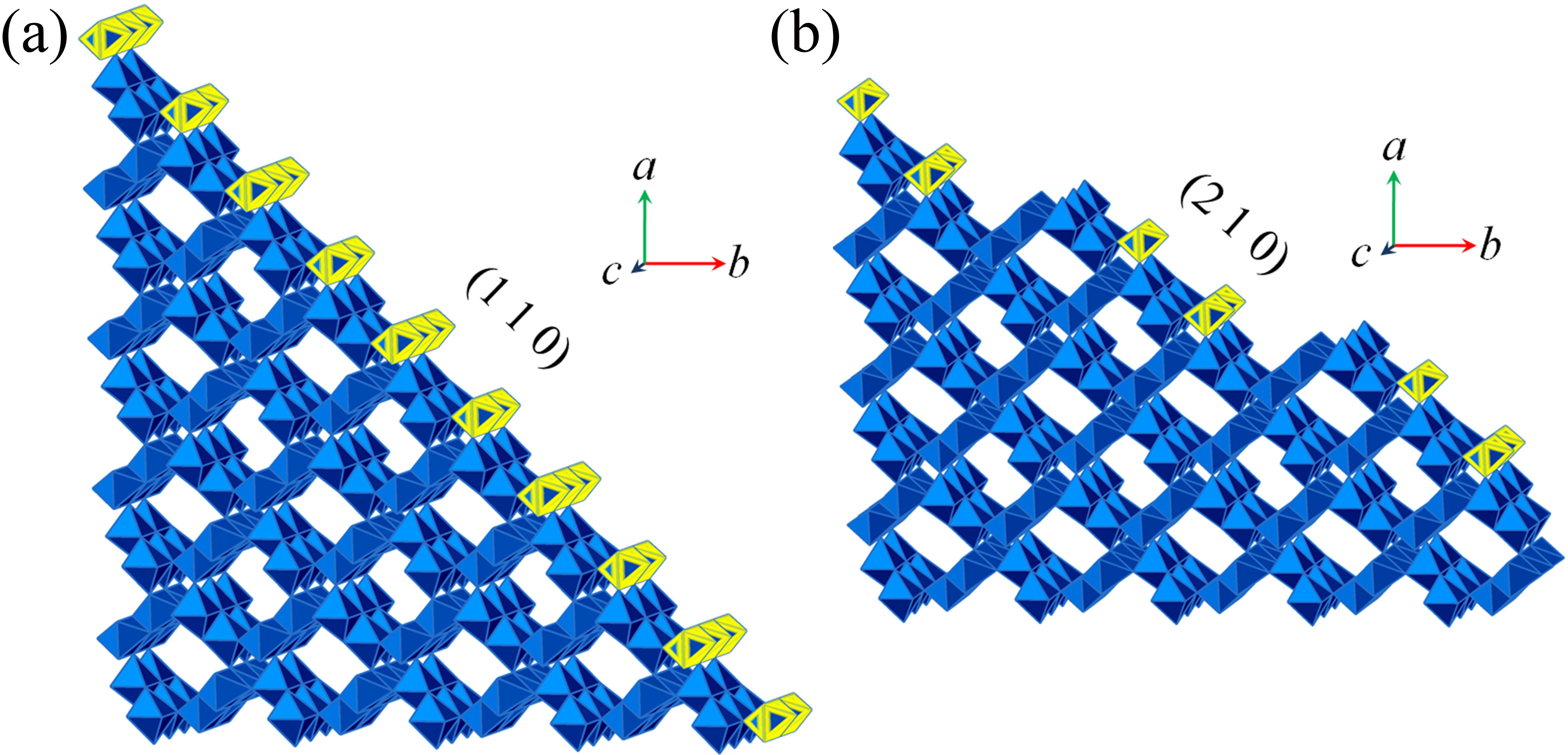


**Figure S5** The crystal structure of the outmost layers for α-MnO_2_ with exposed (1 1 0) and (2 1 0) plane. The highlighted MnO_6_ octahedra hinder the exchange process of Li^+^ ions between electrolyte and the (2 × 2) tunnels in α-MnO_2_ lattice.
